# Effectiveness of the Lower Eyelid Suspension Using Fascia Lata Graft for the Treatment of Lagophthalmos due to Facial Paralysis

**DOI:** 10.1155/2015/759793

**Published:** 2015-03-04

**Authors:** Selam Yekta Sendul, Halil Huseyin Cagatay, Burcu Dirim, Mehmet Demir, Zeynep Acar, Ali Olgun, Efe Can, Dilek Guven

**Affiliations:** ^1^Department of Ophthalmology, Sisli Hamidiye Etfal Training and Research Hospital, Etfal Street, Sisli, 34371 Istanbul, Turkey; ^2^Department of Ophthalmology, Faculty of Medicine, Kafkas University, Pasacayiri Street, 36301 Kars, Turkey

## Abstract

*Purpose*. To evaluate of functional and cosmetic effectiveness of lower eyelid sling technique with fascia lata graft in patients with lagophthalmos due to facial paralysis. *Material and Method*. Ten patients with a mean age of 55.1 ± 19.77 years who underwent lower eyelid sling surgery with a fascia lata graft between September 2011 and January 2014 were included in this prospective study. Preoperatively and postoperatively patients were evaluated in terms of corneal epithelial defects, Schirmer's test, and tear break-up time (TBUT). Cosmetically, vertical eyelid aperture, margin reflex distances 1 and 2 (MRD1 and MRD2) and scleral show were evaluated preoperatively and postoperatively. *Results*. One patient had facial paralysis on the right side whereas the other 9 patients had facial paralysis on the left side. Preoperatively, 3 patients were detected with corneal ulcer, whereas 7 patients were detected with persistent corneal epithelial defects localized in the lower half of the cornea. In the 3 patients with preoperative corneal ulcer, the ulcer recovered with corneal opacity, whereas in the 7 patients with punctate epitheliopathy, postoperative corneal transparency was obtained. *Discussion*. Lower eyelid sling technique with fascia lata graft is an effective technique for the repositioning of the lower eyelid and preventing the corneal complications.

## 1. Introduction

Facial paralysis may occur due to traumatic, viral, iatrogenic, or idiopathic reasons [1, 2]. It may cause permanent complications especially in traumatic or iatrogenic cases [2, 3]. Such cases potentially develop destructive ophthalmic damage such as exposure keratopathy, corneal ulcer, and decrease in vision. The initial aim in all treatments for facial paralysis developed so far is to protect the eye and secondly to achieve the best possible aesthetic appearance. Therefore, in facial paralysis, many surgical techniques such as temporary or permanent tarsorrhaphy, upper eyelid loading with gold or platinum implant, lower eyelid lateral canthal sling, and midface lift have previously been tried and the results have been discussed [2, 4–6].

All facial and periorbital muscles that are innerved by the facial nerve are severely affected by facial paralysis. The most important structures that are not affected by facial paralysis in the facial region are the bone structure, periosteum and canthal bonds, and tarsal structure that form the eyelid skeleton. In this study, the effectiveness of lower eyelid sling technique with fascia lata graft in the prevention of eye surface pathologies due to facial paralysis as well as cosmetic appearance and durability thereof and the results achieved will be discussed.

## 2. Material and Method

The study was planned as a prospective one involving at least 10 patients. Patients who were admitted to our clinic with lagophthalmos due to facial nerve paralysis and who had ocular surface pathologies were randomly included. Patients who previously underwent gold weight implantation surgery due to lagophthalmos or underwent other periorbital surgeries for these reasons and who currently did not have ophthalmic complaints and were not detected with ocular surface pathologies were excluded. Patients who developed gold implant extrusion, or, despite the eyelid loading, who have ocular surface pathologies and complaints were included in this study. All patients underwent a complete ophthalmologic examination preoperatively. All patients preoperative and postoperative Schirmer's test (ST), tear break-up time (TBUT) test, and ocular surface pathologies were evaluated. Again preoperatively and postoperatively, vertical eyelid aperture of both eyes, margin reflex distances 1 and 2 (MRD1 and MRD2), and scleral show (the distance between the corneal limbus and lower lid in primary gaze) were measured. Again, the previous and current daily number of tear drops and gels used for the aim of preventing exposure keratopathy were recorded preoperatively and postoperatively.

The effectiveness of the lower eyelid sling with fascia lata graft was evaluated in terms of function and cosmetics. As the functional success criteria, corneal and conjunctival surface pathologies due to exposure and the daily amount of tear drops and gels used preoperatively were taken as the basis whereas the cosmetic success criteria were quantitative measurements of the eyelid position (vertical eyelid aperture, MRD1, MRD2, and scleral show). The study was conducted in accordance with the tenets of the Declaration of Helsinki by obtaining written consent from all patients, with the approval of the local ethical review board.

### 2.1. Surgical Technique

All patients were operated on under general anesthesia by the same surgeon. Preoperatively the lower eyelid level was arranged to cover the lower limbus while the inner canthal tendon region, lower eyelid 1/2 middle section, and outer orbital wall were marked. Firstly, the anterior leg of the inner canthal tendon was exposed by opening the skin with a vertical incision of approximately 10 mm on the inner canthal tendon. A bed was formed under the anterior leg of the tendon for the passage of the fascia lata band. Then, in the same session, a fascia lata graft of about 3 mm width and 7-8 cm length was taken. The fascia lata graft was passed under the anterior leg of the inner canthal tendon upwards through the previously formed graft bed and was fixed by suturing with a 6/0 prolene suture both the graft itself and the anterior leg of the tendon. Then, a 5 mm length horizontal skin incision was made in the 1/2 middle section of the lower eyelid and it was dissected to the tarsal plate. After that, with the help of a holed hook, a bed was opened for the facial graft over the tars to the inner canthal tendon thereby passing the other leg of the fascia lata and taking it out at the middle 1/2 section of the eyelid. Then, outer canthotomy and cantholysis were made and the dissection was continued until the periost tissue was exposed. A vertical cut was made on the periost on the lateral orbital rim and the periost was dissected so as to expose the lateral orbital bone rim. After the lower eyelid was given the desired position, the lateral orbital bone rim was marked and a hole was formed on the bone by “tour motor.” A bed was formed on the tars laterally for the fascia lata until the middle 1/2 section of the lower eyelid and the fascia lata was taken out from the lateral via a holed hook. At this stage, in patients with eyelid laxity, the eyelid was shortened at an appropriate amount. The lateral end of the fascia lata was taken out from the opened bone inside out and it was fixed in an overlapping fashion by suturing with a 6/0 prolene suture. The periost tissue was closed with a 6/0 vicryl suture. Then, the inner and outer cantus areas were closed firstly in the subcutaneous area with a 6/0 vicryl suture and secondly on the skin with a 6/0 prolene suture. By placing the traction suture (4/0 silk) to the free edge of the lower eyelid, the lower eyelid was lifted to the forehead area (Figures 2(a)–2(e)). For a week, all patients received orally 1000 mg amoxicillin clavulanate potassium two times a day, topical ciprofloxacin pomade once a day, and fluorometholone drops 4 times a day. The traction suture was removed 3 days later whereas skin sutures were removed a week later. Follow-up examinations were made in postoperative day 1, week 1, month 1, and month 3 and the subsequent examinations were planned semiannually. Mean follow-up time was 15.7 ± 9.08 (range 6–34) months.

### 2.2. Statistical Method

SPSS was used for data analysis. In the descriptive statistics of the data, mean, standard deviation, median, minimum-maximum, ratio, and frequency values were used. The distribution of the variables was controlled via Kolmogorov-Smirnov test. In the analysis of quantitative data, sample *t*-test and Wilcoxon test were used.

## 3. Results

Ten patients (4 males, 6 females) with a mean age of 55.1 ± 19.77 (range 19–75) years who underwent lower eyelid sling surgery with a fascia lata graft were included in this study. Nine patients had facial paralysis at the left side whereas 1 patient had facial paralysis at the right side. Two patients had a history of previous cerebellopontine angle tumor operation whereas 2 had trauma, 3 cerebral hemorrhage, 1 previous parotid gland tumor operation, and 2 idiopathic facial paralysis (Table 1). The average period between the facial paralysis occurrence and the surgery was 4.9 (range: 2 to 10) years. In addition to eyelid laxity and retraction, 6 patients had ectropion as additional pathology. As ocular surface pathology, 3 patients had deep corneal ulcer in the lower half of the cornea whereas the rest of the patients had corneal epitheliopathy shown in fluorescein paint (Table 2). Moreover, all patients had conjunctival hyperemia in various degrees. Two patients had previously undergone gold weight implantation surgery and 2 patients had a history of temporary tarsorrhaphy whereas the remaining 6 patients had no periorbital surgery history.

The results were collected under two categories, that is, functional and cosmetic. Functionally, preoperative and postoperative Schirmer's test, TBUT measurements, corneal surface changes, and the daily use of tear drops and gels were evaluated. In terms of Schirmer's test, the difference between the paralytic side and the healthy side in the preoperative and postoperative measurements was not significant (*P* > 0.05) whereas at the paralytic side, there was significant increase in postoperative Schirmer's measurement results compared to that of the preoperative measurement (*P* < 0.05). In terms of tear break-up time (TBUT) in the eye at the paralytic side, preoperative and postoperative TBUT values were significantly lower compared to the healthy eye (*P* < 0.05). Again, in the eye at the paralytic side, postoperative TBUT value increased significantly compared to the preoperative TBUT value (*P* < 0.05) (Figure 3). In the 3 patients with preoperative corneal ulcer, the ulcer recovered with corneal opacity whereas in the 7 patients with punctate epitheliopathy, postoperative corneal transparency was obtained. Another important point was the amount of tear drops and gels used by the patient daily. In the eyes at the paralytic side, preoperative daily use rate of tear drops and gels was an average of 17.6 ± 3.5 whereas this rate dropped to 3.0 ± 1.1 postoperatively.

Cosmetically vertical eyelid aperture, MRD1, MRD2, and scleral show measurements were evaluated. No significant difference was found in terms of vertical eyelid aperture between the paralytic side and the healthy side preoperatively and postoperatively (*P* > 0.05). In the eye at the paralytic side, even though the postoperative measurement showed a decrease of about 1 mm in the vertical eyelid aperture compared to that of the preoperative measurement, this change was not statistically significant (*P* > 0.05). Another measurement compared was the MRD1 value. No significant difference was found between the preoperative and postoperative MRD1 values of the paralytic eye and the healthy eye (*P* > 0.05) and again in paralytic eyes, no significant change was found preoperatively and postoperatively (*P* > 0.05). Another important value that we compared was the MRD2 value. In the eyes at the paralytic side, the preoperative MRD2 values were significantly higher than that of the eyes at the nonparalytic side (*P* < 0.05). Again, similarly, in the paralytic eyes, the postoperative MRD2 values were significantly lower than the preoperative values (*P* < 0.05). In terms of postoperative MRD2 values, there was no significant change between the two eyes while in the eyes at the paralytic side, lower eyelid was raised (*P* < 0.05). Perhaps the most important and valuable measurement of this study was the scleral show. In this regard, in the eye at the paralytic side in comparison to the healthy side, preoperative scleral show measurement was significantly higher (*P* < 0.05). With respect to the eye at the paralytic side, the scleral show distance was significantly lower postoperatively in comparison to the preoperative value (*P* < 0.05) (Figure 4). Again, a postoperative comparison of the healthy and the paralytic eyes revealed no significant changes and the lower eyelid at the paralytic side was raised (*P* > 0.05) (Table 3).

Four patients who developed eyebrow ptosis due to facial paralysis underwent direct browpexy as secondary surgery whereas one patient with ongoing postoperative punctum eversion and eyelid ectropion underwent punctum eversion correction surgery as secondary surgical intervention. In one patient, wound infection developed in the leg (fascia lata harvested area) whereas one patient developed suture reaction in the medial canthal area. These infections were treated with systemic antibiotherapy. Other than that, no complications were observed.

## 4. Discussion

Many changes occur in the face and also in the periorbital area after facial paralysis. After a long-term paralysis, especially with the influence of gravity, changes may be observed that are likely to seriously affect the eye surface such as laxity in the lower eyelid, ptosis and ectropion [7]. Regarding irreversible facial paralysis, both dynamic and static surgical methods have been tried in treatment of the paralytic lagophthalmos, and their results have been discussed [6, 8–13]. In this study, we started from questions as to whether through surgical intervention of only the lower eyelid the eye can be effectively protected from problems caused by exposure and also whether in performing this operation more aesthetical and constant results can be achieved.

**Figure 1:**
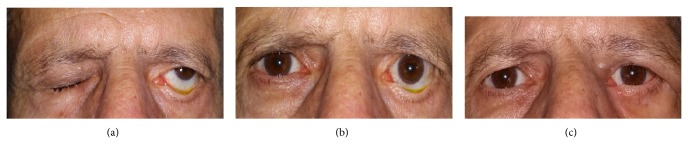
(a) Male patient aged 55, with lagophthalmos, lower eyelid ptosis, and ectropion in the lower eyelid due to facial paralysis 5 years ago. (b) Fornix insufficiency and location of the tear meniscus at the inferior-posterior level due to distinct lower lid margin-limbus and ectropion. (c) The same patient at the postoperative month 3. Lower lid margin-limbus distance zero (positive cornea transparency and negative fluorescein uptake in the slit lamp examination).

**Figure 2:**
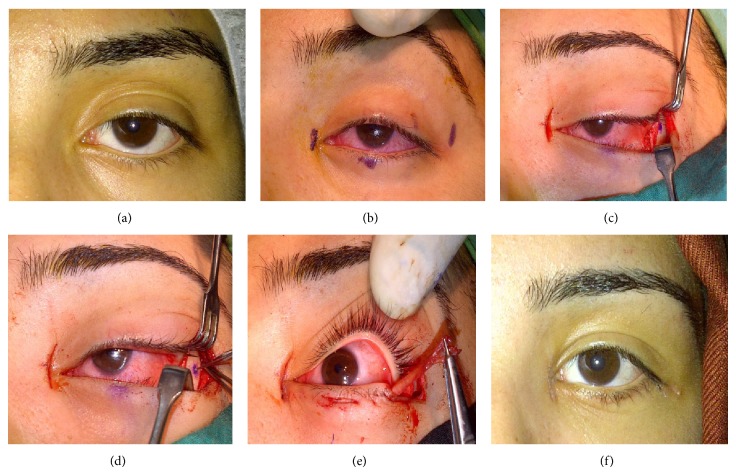
(a) Female patient aged 19, with lower eyelid retraction and increased sclera show distance following facial paralysis on the left side (scleral show: lower lid margin-limbus). (b) Lower eyelid marked according to the desired postoperative shape. (c) With medial and lateral incisions, the anterior leg of the inner canthal tendon at the medial and lateral orbital bone rim at the lateral was exposed. (d) A hole was formed on the lateral orbital bone wall by tour motor. (e) Fascia lata fixed on the inner canthal tendon was passed through the bed formed on the lower lid tars and brought to the lateral. Then, it was passed through the hole on the lateral orbital bone and fixed. (f) The same patient at postoperative month 6. Lower lid margin-limbus distance zero.

**Figure 3:**
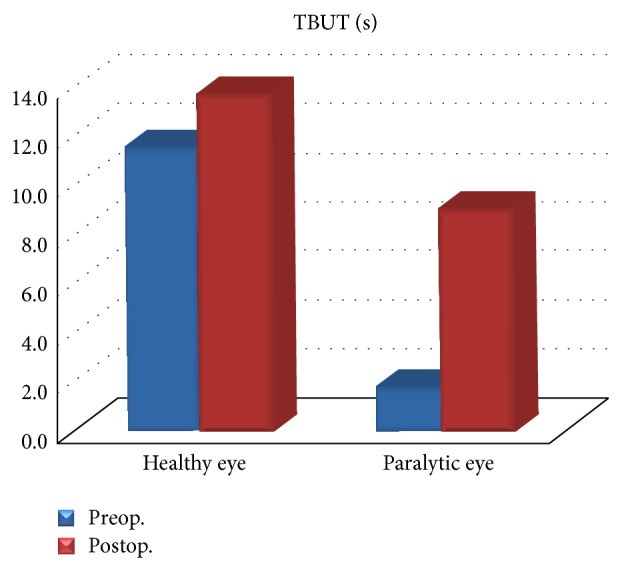
Preoperative and postoperative TBUT rates. Significant increase of the postoperative TBUT rate in the paralytic eye.

**Figure 4:**
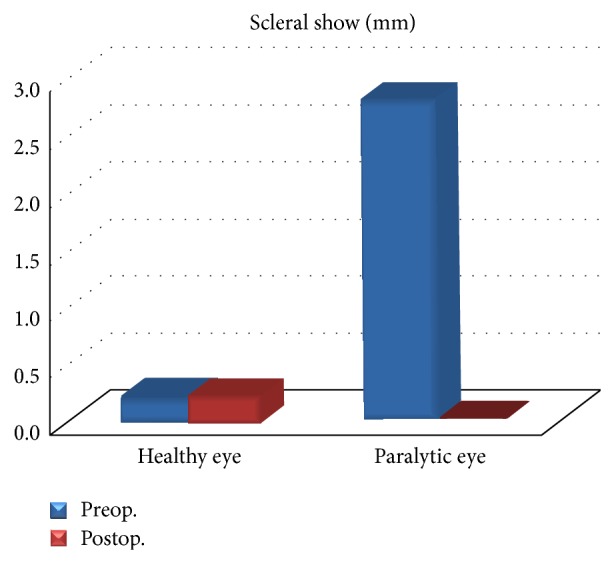
Note the postoperative increase in the distance between the lower lid margin and the lower limbus.

In terms of its functional effects on the periocular area, facial paralysis mostly affects the lower eyelid. That is, after a long-term paralysis, the orbicular muscle that surrounds the eye faces serious tonus loss and considering the additional effect of gravity, the lower eyelid sags downwards. The lower eyelid that sags downwards moves away from the convex corneal surface of the globe in the inferior-posterior direction and causes pseudoproptosis appearance in the globe. On the other hand, the lower eyelid that moves away from the corneal surface also causes decrease in the lower fornix volume that somewhat constitutes a tear pool for the eye surface. Due to all these reasons, tears spontaneously flow outwards because the fornix volume is decreased and the lower eyelid is moved in the inferior-posterior direction and has experienced contraction loss. In other words, tear meniscus that is formed in normal eyes at the junction of the lower eyelid edge and the lower limbus, is now formed weakly at a lower point, that is, in the junction of the sclera and the lower eyelid edge (Figures 1(a)–1(c)). We believe that the greatest evidence to this situation is the beginning of the formation of exposure keratopathy in the lower half of the cornea. Another problem is that due to the contraction loss in the orbicular muscle, lacrimal pumping system is subject to damage. As a result of all these pathological processes, ectropion is eventually added into the pathological process due to the tear flowing downwardly and outwardly, the behavior of the patients (wiping the tear away), and the environmental conditions.

Many methods such as metallic palpebral bow implant and subcutaneous elastic silicon implant have been developed in order to enable the paralytic eyelid to close dynamically [14]. On the other hand, combined dynamic and static closure of the eyelid may be obtained by temporal fascia transposition procedure. Miyamoto et al. [15] reported that with temporal muscle transfer, they obtained total closure in the eyelids at a rate of 78.75% and a success rate of 60% in other symptoms such as dry eye and pain. Ueda et al. [16] in their comparative study in which they applied gold weight on the upper eyelid with temporal fascia transposition reported that although the two methods were similar in terms of symptom relief, the closure of the eyelid improved with temporal fascia transposition. However, they also added that one important disadvantage of temporal fascia transposition was unwanted closing of the eye during eating.

The most common static surgical treatment on the paralytic eyelid is the placement of weight load on the upper eyelid. Ever since the study of Smellie [8] in 1966 reporting a single case that underwent gold weight implantation, gold weight implantation has been affected by different techniques and many studies and their results have been reported [6, 9–11]. What is deduced from these studies is that the most important problems of gold weight implantation are extrusion, migration, ptosis, and reaction to the implant though partially and ongoing eyelid edema. In a study in which flexible chain platinum implant and rigid gold implant were compared, it was reported that flexible chain platinum implant gave more effective results and caused less complications [5].

The most important way to protect the ocular surface is obviously eliminating the ocular area that is exposed to the outer world or at least lessening it. In this regard, gold weight implants and similar surgical techniques are rational leaving aside complications and cosmetically bad appearance; however, admittedly, they are not single handedly effective because another factor as important as eliminating the ocular area that is exposed to the outer world is the dynamic movement of the tear that protects and feeds the ocular surface. The most important aspect that provides this movement to the tear is the dynamism of the eyelids. Therefore, is it possible to redistribute the tear to the ocular surface in paralytic eyes that lose eyelid dynamism or could this be at least partially achieved? Our aim was to decrease the ocular surface that is exposed to the outer world by slinging the lower eyelid in a balanced way and at the same time, through this operation, to recreate the tear pool in the lower fornix and the tear meniscus that moved away from the cornea. Terzis and Kyere [17] in their retrospective comparative broad study of 20 years which is similar to our study applied lower eyelid sling with only minitendon obtained from palmaris longus tendon or minitendon/gold weight or minitendon/eye spring and reported that the lower eyelid sling was effective in terms of scleral show, that there was no statistical difference between the groups, and that minitendon is reliable regardless of age and paralytic process. Again, in a similar method, another study in which tendon and lower eyelid are secured to the periorbital bone tissue with screw at the lateral and medial side reported that this method is effective for lagophthalmos [18].

Surgical treatments of the lower eyelid in facial paralysis such as lateral canthal sling, lateral tarsal strip, and suborbicularis oculi fat (SOOF) lift have usually been given to patients that developed lower eyelid ectropion or eyelid retraction and have been reported to be effective [14, 19]. These treatments have generally been auxiliary to weight loading treatments to the upper eyelid or supplementary treatments. In this study we aimed to correct the functional and cosmetic status of the lower eyelid with an effective surgery which did not include the upper eyelid. Furthermore we avoided the upper eyelid loading surgeries which may cause the ptosis. Four patients who developed eyebrow ptosis due to facial paralysis underwent direct browpexy as secondary surgery and obtained better cosmetic results. Again as an auxiliary technique in patients with lower eyelid retraction, hard palate and cartilage grafts were used as rear lamellar grafts in order to lift the lower eyelid [20]. An interesting study by Goldberg et al. [21] involves enlarging the lower eyelid by hyaluronic acid gel. The authors reported that it is indeed a preferable alternative especially for patients who had undergone multiple operations as it is a temporary approach that requires minimal invasion and also carries the advantage of filling the periorbital holes.

The success of a surgical treatment depends on the ease of performance, short surgical time, and long-term effectiveness. In our study, the minimum follow-up time was 6 months whereas the maximum follow-up time was 34 months. Within this period, none of the cases showed a recurrence of ectropion, and none of the cases showed damage due to exposure in the ocular surface. Postoperatively, no complication detected such as exposure of the suspension material and scar formation except one patient who developed suture reaction in the medial canthal area. We think that it is related to the high biocompatibility of the suspension material and easy integration of the periocular tissue. However, this is not a sufficient term for assessing the long-term effectiveness of surgical success and for this aim new supporting studies must be conducted. Other significant drawbacks of this method are long surgical time and particularly the damage that is likely to be made to the lacrimal system while working on the medial canthal area. Another limitation of this study was a few number of patients. Yet, the study has the major advantages of being prospective and showing the effectiveness of ocular surface protection factors through quantitative measurements.

## 5. Conclusion

The lower eyelid sling technique with fascia lata, despite its long surgical time, is an effective and alternative method of treatment for both ocular surface protection and cosmetic appearance.

## Figures and Tables

**Table 1:** Etiology.

	*n*
(1) Previous cerebellopontine angle tumor surgery	2
(2) Trauma	2
(3) Cerebral hemorrhage	3
(4) Parotid gland tumor surgery	1
(5) Idiopathic	2

**Table 2:** Epidemiological data of patients and pre/postoperative ocular surface findings. (Med: Median, Min: minimum, Max: Maximum, Avg: Average, S.d: Satandart deviation).

	Med (Min–Max)	Avg. ± s.d./(*n*-%)
Age	62.5 (19–75)	55.1 ± 20.8
Gender		
Female		6 (60.0%)
Male		4 (40.0%)
Preoperative number of drops (×2)	17.0 (12–24)	17.6 ± 3.5
Postoperative number of drops (×1)	3.0 (2–4)	3.0 ± 1.1
Pre/postoperative		
Corneal ulcer	Opacity	3 (30.0%)
Corneal epitheliopathy	Transparent	7 (70.0%)

**Table 3:** Statistical values of preoperative and postoperative Schirmer, TBUT, PA, MRD1, MRD2, and scleral show distances (TBUT: tear break-up time, PA: palpebral aperture, MRD1: margin reflex distance 1, MRD2: margin reflex distance 2, and scleral show distance: lower lid margin-limbus distance). (Med: Median, Min: minimum, Max: Maximum, Avg: Average, S.d: Satandart deviation).

	Healthy eye		Paralytic eye		*P*
	Avg. ± s.d.	Med (Min–Max)	Avg. ± s.d.	Med (Min–Max)	
Schirmer's (mm)					
Preoperative	15,1 ± 9,2	15,0 (3–30)	10,6 ± 5,8	8,5 (3,0–20,0)	0,240
Postoperative	14,2 ± 7,8	15,0 (3–31)	18,3 ± 5,3	17,0 (10,0–25,0)	0,196
Pre/postoperative change *P*	0,766		**0,000**		****
TBUT (s)					
Preoperative	11,5 ± 2,1	11,0 (9–15)	1,8 ± 1,3	2,0 (0,0–4,0)	**0,005**
Postoperative	13,7 ± 5,9	12,0 (10–30)	9,0 ± 2,6	8,5 (6,0–14,0)	**0,013**
Pre/postoperative change *P*	0,473		**0,005**		****
PA (mm)					
Preoperative	8,6 ± 1,4	9,0 (6–11)	8,8 ± 1,1	9,0 (7,0–11,0)	0,343
Postoperative	8,5 ± 1,2	8,5 (6–10)	8,1 ± 1,5	8,0 (5,5–10,0)	0,287
Pre/postoperative change *P*	0,780		0,264		****
MRD 1 (mm)					
Preoperative	3,0 ± 0,7	3,0 (2–4)	3,1 ± 0,8	3,0 (2,0–5,0)	0,541
Postoperative	3,1 ± 0,7	3,0 (2–4)	3,0 ± 0,9	3,3 (2,0–4,0)	0,678
Pre/postoperative change *P*	0,193		0,642		****
MRD 2 (mm)					
Preoperative	5,3 ± 0,8	5,3 (4–6)	8,2 ± 1,4	8,0 (5,5–10,0)	**0,000**
Postoperative	5,4 ± 0,7	5,3 (4–6)	5,1 ± 0,8	5,0 (3,5–6,5)	0,279
Pre/postoperative change *P*	0,443		**0,000**		****
Scleral show (mm)					
Preoperative	0,2 ± 0,4	0,0 (0-1)	2,8 ± 0,9	3,0 (1,0–4,0)	**0,005**
Postoperative	0,2 ± 0,4	0,0 (0-1)	0,0 ± 0,0	0,0 (0,0-0,0)	0,157
Pre/postoperative change *P*	1,000		**0,004**		****

Matched sample *t* test/Wilcoxon test.
